# Evolving Medical Students’ Digital Health Perceptions and Intentions: Insights From a Prepandemic and Postpandemic Survey Study

**DOI:** 10.2196/64804

**Published:** 2025-09-03

**Authors:** Mickaël Ringeval, Louis Raymond, Marie-Pascale Pomey, Guy Paré

**Affiliations:** 1 Department of Computer Information Systems, Bentley University, 175 Forest Street, Waltham, MA, 02452, United States, 1 7818910000; 2 Université du Québec à Trois-Rivières, Trois-Rivières, QC, Canada; 3 Carrefour de l’Innovation, Centre de recherche du Centre hospitalier de l'Université de Montréal, Montréal, QC, Canada; 4 Evaluation et politique de santé, école de santé publique, Département de gestion, Université de Montréal, Montréal, QC, Canada; 5 Centre d’excellence sur le partenariat avec les patients et le public, Montréal, QC, Canada; 6 Research Chair in Digital Health, HEC Montréal, Montréal, QC, Canada

**Keywords:** digital health, eHealth, medical education, medical practice, artificial intelligence, COVID-19, survey

## Abstract

**Background:**

Digital health (dHealth) technologies, such as telehealth, artificial intelligence (AI), and mobile apps, are increasingly essential in medical practice. However, despite their growing significance, medical curricula often lack structured dHealth training, leaving students underprepared for digitally integrated health care environments.

**Objective:**

This study investigates the factors influencing medical students’ intentions to integrate dHealth technologies into their future practice and examines changes in their perceptions over time.

**Methods:**

We conducted a 2-phase survey at a large Canadian medical school to assess changes in perceptions before (N=184) and after (N=177) the COVID-19 pandemic. A mixed methods approach combined component-based structural equation modeling and fuzzy-set qualitative comparative analysis. The model was grounded in the technology acceptance model and Triandis’ theory of interpersonal behavior, examining constructs such as individual background, facilitating conditions, perceived usefulness, and beliefs about AI.

**Results:**

Across both phases, over 85% (306/361) of students agreed that dHealth education should be a mandatory component of medical training. Mean ratings for intention to use dHealth in future practice increased significantly between t_0_ and t_1_ for patient communication (3.4 to 4.2, *P*<.001), monitoring (3.3 to 4.0, *P*<.001), and diagnosis/treatment (3.6 to 4.2, *P*<.001). Experience with AI tools increased from 1.3 to 1.5 (*P*<.001), and telehealth from 1.2 to 1.6 (*P*<.001), while exposure to hospital IT systems and mobile apps remained unchanged. Results confirmed that perceived usefulness (*β*=.37 at t_0_; *β*=.34 at t_1_) and beliefs about AI (*β*=.39 at t_0_; *β*=.27 at t_1_) were strong predictors of intention to integrate dHealth (*P*<.001). The explanatory power of the structural equation modeling model declined postpandemic (*R*²=0.53 at t_0_ vs *R*²=0.25 at t_1_), suggesting increasing complexity in influencing factors. Fuzzy-set qualitative comparative analysis revealed multiple configurations leading to high intention, with consistency values exceeding 0.88 and overall solution coverage of 0.74 postpandemic. Core conditions across high-intention profiles included strong beliefs in the role of AI and perceived importance of dHealth education. Conversely, gender appeared as a recurring core condition in non–high-intention configurations, suggesting persistent disparities in dHealth adoption.

**Conclusions:**

The study advocates for the integration of formal dHealth training in medical curricula to better prepare future physicians for the demands of an increasingly digital health care landscape. While the COVID-19 pandemic may have contributed to shifting perceptions, other factors, such as recent AI advancements, likely played a role. These findings highlight the urgent need for medical education to adapt to the changing dHealth environment.

## Introduction

### Background

Digital health (dHealth) is broadly defined as the use of digital technologies and information systems (ISs) to enhance the delivery of health care and improve health outcomes [1]. This notion encompasses a wide range of technologies and apps that aim to enhance health care services, monitor health conditions, promote wellness, and facilitate communication between patients and health care providers. Over the past decade, dHealth has attracted a great deal of attention and has been at the forefront of major digital transformation initiatives [2,3]. Indeed, the advent of dHealth technologies has transformed the health care landscape, converting it into a dynamic industry, with market projections soaring to over US $270 billion by 2028 [4].

The growing significance of dHealth technologies stems from their potential to optimize health care delivery, empower patients, and extend the reach of care, particularly among older and vulnerable populations [5,6]. These tools have played a critical role in enhancing care quality, continuity, and accessibility, especially during the COVID-19 pandemic, which accelerated the mainstream adoption of web-based platforms, teleconsultations, and artificial intelligence (AI) tools [7,8]. As a result, future physicians are increasingly expected to demonstrate digital competencies, including navigating electronic health records (EHR), maintaining data privacy, communicating effectively in remote settings, and understanding telehealth regulations.

Despite these trends, the integration of dHealth into undergraduate medical education remains uneven. While the extant literature recognizes the importance of equipping future clinicians with digital competencies [9-11], many medical schools have been slow to incorporate dHealth training into their formal curricula. This limited exposure may hinder students’ ability to navigate digital environments and may even lead to unintended consequences, such as breaches in patient confidentiality. Although several studies have identified institutional and curricular barriers to dHealth integration [12,13], much less attention has been paid to medical students’ own perspectives, intentions, and perceived preparedness.

Furthermore, previous research has typically examined isolated technologies (eg, AI in radiology [14,15], EHRs [16], or telemedicine [17]) or local contexts (eg, studies conducted in Canada [14], the United Kingdom [15], Vietnam [18], or Finland [19]), offering fragmented insights into how students engage with dHealth more broadly.

To address the abovementioned gaps, this study investigates the determinants of medical students’ intention to use dHealth technologies in their future practice. Uniquely, it adopts a 2-phase design comparing data collected before and after the COVID-19 pandemic, a period that marked a turning point in dHealth adoption. Accordingly, we pose the following research questions: (1) How inclined are medical students to integrate dHealth technologies into their future practice? (2) What factors influence their intentions? (3) How have medical students’ attitudes and intentions toward dHealth changed between the prepandemic and postpandemic periods?

In answering these questions, the study moves beyond attributing changes solely to the pandemic. It recognizes that shifts in students’ intentions may also reflect broader transformations, such as the emergence of new digital tools and recent efforts to modernize medical curricula. By offering a temporally comparative analysis, this study provides timely insights for educators, policymakers, and curriculum developers seeking to prepare the next generation of physicians for digitally enabled care.

### Theoretical Model and Hypotheses

To address our research questions, we begin by developing a theoretical model with 2 main foundations. The first theoretical component is the technology acceptance model (TAM), including all its variants. The TAM provides a conceptual framework to explain how users come to adopt a new technology [20]. Building on prior research on dHealth training [15,16] and the vast body of literature on the TAM [17,18], we adopt Individual Background as a generic construct in our model. This construct is operationalized as a composite of 3 attributes: gender, age, and academic level. Given the exploratory nature of our study, we postulate that individual background might exert an influence on students’ beliefs and attitudes toward dHealth technologies. This forms the basis for Hypotheses 1 and 2.

According to the TAM, Facilitating Conditions are likely to influence medical students’ behavioral intentions. Indeed, Facilitating Conditions influence an individual’s perceptions of how difficult the performance of a certain task is [21-23]. In this study, Facilitating Conditions are operationalized as students’ prior experimentations with dHealth technologies during their academic journey. We first posit that increased exposure of medical students to dHealth technologies within medical schools enhances their understanding of the benefits and capabilities of such innovations. Consequently, this heightened comprehension is expected to foster a greater perception of usefulness. Similarly, expanded opportunities for medical students to engage with dHealth technologies, and with AI apps in particular, are anticipated to bolster their beliefs in the positive impact of AI on the medical field and their own medical practice. Finally, we posit that a higher level of experience with dHealth technologies during their medical education correlates with a greater intention among students to integrate dHealth technologies into their future practice. These propositions align with Hypotheses 3, 4, and 5.

As mentioned above, our model includes Perceived Usefulness, another important component of the TAM. It is broadly defined as the extent to which individuals believe that the adoption of a technology would improve their performance. In this study, Perceived Usefulness is adapted to refer to medical students’ perceptions of the relevance of integrating dHealth into the medical curriculum. Hypothesis 6 posits that the stronger the belief of medical students that they should receive dHealth training as an integral part of their medical training, the more likely it is that they will have the intention to incorporate dHealth technologies into their future medical practice.

**Figure 1.**
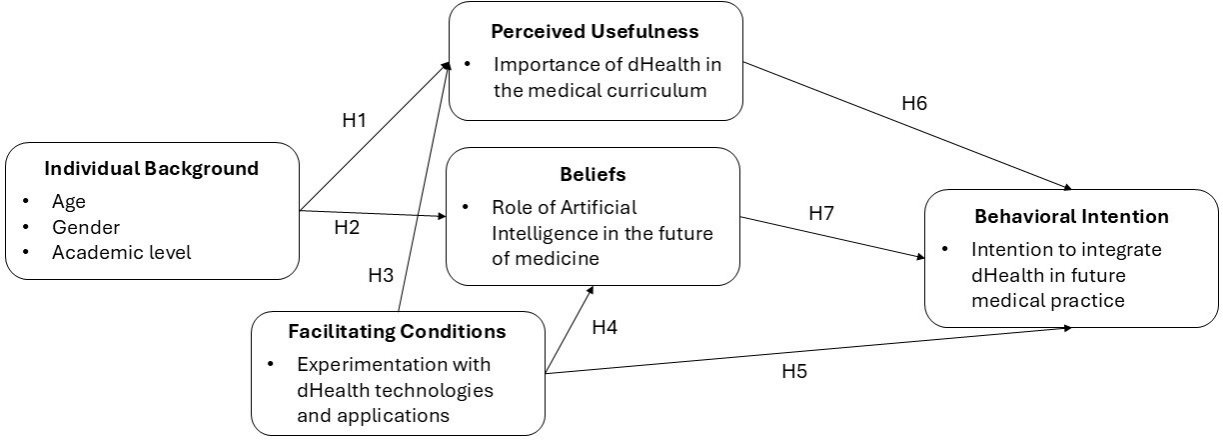
Theoretical model. dHealth: digital health.

Our second theoretical foundation is Triandis’ theory of interpersonal behavior [24]. This theory posits that the behavioral intentions of individuals are shaped by their beliefs about the behavior. Triandis defines belief as an individual’s assessment of an object of interest. It is important to note that for Triandis, belief is not necessarily shaped by value judgements about the “goodness” or “badness” of the object of interest. Instead, it consists of assessments of the existence or absence of certain attributes. For example, an individual might hold the belief that, as a general rule, technology contributes to the advancement of society. In our study, we operationalized medical students’ beliefs about AI-related technologies specifically, rather than beliefs involving a broader assessment of dHealth technologies. The specificity of this operationalization stems from the recognition that AI-based tools hold immense potential to transform and enhance the practice of medicine [25,26]. Hypothesis 7 posits that the more medical students believe in the positive impact of AI on the field of medicine and on their own medical practice, the more likely it is that they will have the intention to integrate dHealth technologies into their future medical practice. The theoretical model is shown in Figure 1.

## Methods

### Setting and Participants

In Canada, where the study was conducted, medical education typically consists of a 4- or 5-year undergraduate medical program, depending on the institution, followed by a residency period that varies in length based on the chosen specialty. Medical programs generally admit students after completing either a bachelor’s degree or a minimum number of undergraduate credits, with some institutions offering direct-entry programs from high school. The first 2 years of medical education are typically preclinical, focusing on foundational biomedical sciences, while the subsequent years involve clinical training through clerkships in hospitals and community settings. Following medical school, graduates enter a residency program accredited by the Royal College of Physicians and Surgeons of Canada or the College of Family Physicians of Canada, which lasts between 2 and 7 years, depending on the specialty.

This study was conducted at a large medical school in Quebec province. The curriculum at the participating institution follows the general structure of Canadian medical education, with preclinical years emphasizing foundational sciences and clerkship years providing hands-on clinical experience. Notably, the standard curriculum does not include formal dHealth training. However, students have access to a web-based training platform that offers educational content on various subjects, including clinical ISs. Additionally, workshops on mobile health and electronic medical records are mandatory during clerkship, a stage where students gain practical experience and have opportunities to engage with different dHealth technologies. Furthermore, the medical school addresses topics related to the professional use of social media, email, and mobile apps in elective seminars.

The study population consisted of 1462 medical students. It comprised 2 phases of a survey questionnaire: an initial survey (t_0_) in February 2020, conducted before the COVID-19 pandemic, and a replication survey (t_1_) in May 2023, which took place after the pandemic. The survey was distributed to all students through the medical school’s mailing list and was also promoted by the medical student association via its newsletter. There was no incentive for students to fill out the web-based questionnaire, and there were no negative consequences if students did not participate. The survey was developed and administered using the Qualtrics survey platform. The study design and survey instrument were approved by the medical school’s ethics committee.

### Questionnaire Development

No existing questionnaire fully captured the variables included in our research model. Therefore, we adapted survey items from previously validated instruments in related contexts [17], modifying them to better align with our study’s focus. Our survey underwent multiple rounds of refinement, further developing the survey items at each round to ensure alignment with established measures. The final instrument was validated through assessment by a panel of 10 medical students, who were not included in the main study sample. The final survey questionnaire consisted of 70 five-point Likert questions and 8 yes/no items and is available in Multimedia Appendix 1.

### Data Analyses

Burton-Jones et al [27] and Levallet et al [28] emphasize the value of integrating complementary research methods, analytic approaches, and theoretical perspectives to better understand ISs phenomena. Our study considers both “variance” and “systems” perspectives. The variance perspective focuses on individual variables, while the systems perspective considers the holistic interplay of factors, excluding the “process” perspective due to its irrelevance to our conceptualization [27]. We applied correlational approaches using component-based structural equation modeling (SEM) and configurational approaches using fuzzy-set qualitative comparative analysis (fsQCA) as outlined by Levallet et al [28]. To ensure consistency, we gathered data via a survey methodology, allowing us to apply both analytical approaches to the same dataset. Adopting a methodological approach, analytic approaches, and theoretical perspective approach, we recognized that our study’s conceptualization of dHealth adoption could be examined from either a variance perspective, which focuses on the impact of individual factors, or a systems perspective, which considers how multiple conditions interact to shape students’ intentions. Following Levallet et al [28], SEM was used to assess the independent effects of key factors in our model, while QCA enabled an exploration of different combinations of conditions that collectively influence students’ dHealth adoption. By applying both approaches to a common dataset collected through survey methodology, we ensured a more comprehensive evaluation of the model.

Using both SEM and QCA offers a more complete understanding of what shapes students’ adoption intention. SEM helps us examine how individual determinants, such as perceived usefulness and facilitating conditions, directly influence intention. In contrast, QCA looks at how different combinations of factors work together to produce high or low intentions [29,30]. This dual approach allows us to capture both the specific effects of single factors and the broader patterns that emerge when multiple influences interact. The following section details the application of these 2 analytical approaches in our study.

The survey data were first analyzed with descriptive statistics and *t* tests. Partial least squares SEM has emerged as a prevalent tool for examining exploratory structural models akin to ours. In this study, we used SmartPLS (version 4*;* Smart PLS GmbH) to test our model [31]. To ascertain the significance levels of the various structural models, we adopted a bootstrapping method, resampling 10,000 times as per the guidelines delineated by Hair et al [32]. This approach entailed the random selection of subsamples, facilitating robust testing of model parameters.

We then analyzed the same dataset with fsQCA, using the “QCA” package on the R platform (version 4.2.3, R Core Team), which involves 3 steps: data table construction, truth table construction, and logical reduction [33]. First, raw data is calibrated into set membership scores. The truth table then shows configurations and their outcomes, which are logically reduced to simplify the solution. Consistency and coverage are key concepts in fsQCA evaluation. Consistency measures how well a condition or combination of conditions predicts an outcome, ranging from 0 to 1. We used a raw consistency threshold of 0.80, a frequency threshold of 3, and a proportional reduction in inconsistency threshold of 0.50 [34]. Coverage indicates the empirical relevance of a configuration, also ranging from 0 to 1. Consistency is akin to significance in correlational analysis, while coverage is akin to *R*^2^. Conditions can be “core” (stronger causal relationship) or “peripheral” based on their impact on the outcome.

Measurement of research variables through a self-administered questionnaire by a single respondent may pose a risk of common method bias (CMB) [35]. We thus chose different question formats and scale types as an initial precautionary measure. We further looked at the inter-construct correlations at t_0_ and t_1_ to determine if any construct pairs correlated above the 0.90 threshold, as this could signal the presence of CMB [36]. Now, every correlation was found to be well below this threshold. Last, we used Harman’s single-factor procedure to further test for the presence of CMB for all scale variables in the measurement model. As multiple factors emerged from the principal component factorial analysis of the variables, and as no single component accounted for 50% or more of the covariance among these variables, this further suggests the absence of CMB [37].

### Ethical Considerations

This study received ethics approval from the Comité d’éthique de la recherche en sciences de l’éducation et de la santé (CERSES) at the University of Montreal, Canada, on October 28, 2019 (certificate number CERSES-19-108-D). The study adhered to the principles of the Declaration of Helsinki, the Committee on Publication Ethics (COPE) guidelines, and all applicable institutional, national, and international ethical standards. Prior to beginning the survey, all participants provided informed consent electronically after being presented with a comprehensive overview of the study’s objectives, procedures, potential risks, and anticipated benefits. No vulnerable populations (such as minors, older adults requiring assistance, or persons with cognitive impairments) were included. No personally identifiable information was collected, and all responses were anonymized before analysis to ensure confidentiality. Throughout the study, participant privacy and data integrity were rigorously protected in line with established research ethics guidelines and applicable data protection regulations. No financial incentives or compensation were offered to participants.

## Results

### Demographic and Descriptive Statistics

It is important to note that no significant modifications were made to the medical curriculum regarding dHealth training during the duration of this study. Hence, the educational opportunities related to dHealth remained consistent across both survey phases.

In the initial survey conducted at time point t_0_, 184 students participated, representing a 13% response rate. At time point t_1_, for the subsequent survey, 177 students responded, accounting for a 12% participation rate. As detailed in Table 1, the majority of participants were women, constituting 65% at t_0_ and increasing to 75% at t_1_. The respondents’ average age was 23 years, aligning with the median age of medical students at the university where the study was conducted. While the survey participants’ distribution across the first to fifth years of medical training was balanced at t_0_, the representation of students in their fourth and fifth years was comparatively lower at t_1_.

**Table 1.** Profile of the respondents.

	Pre–COVID-19, t_0_ (N=184)	Post–COVID-19, t_1_ (N=177)
Academic level, n (%)		
Preparatory year	40 (22)	38 (22)
First year preclinical	36 (20)	75 (42)
Second year preclinical	43 (23)	32 (18)
First year clerkship	33 (18)	18 (10)
Second year clerkship	32 (17)	14 (8)
Gender, n (%)		
Woman	119 (65)	124 (70)
Man	65 (35)	51 (29)
Prefer not to respond	0 (0)	2 (1)
Age (years)		
Mean (SD)	22.9 (3.5)	22.8 (3.3)
Minimum	18	18
Maximum	38	38

### Differences Between Medical Students’ Views and Intentions at t_0_ and t_1_

As shown in Table 2, most medical students reported limited opportunities to engage with dHealth technologies, including hospital IT, telehealth, mobile apps, and AI technologies during their medical education. Second, the overwhelming majority of participants agreed that dHealth education should be a mandatory component of medical training. Medical students prioritized learning about hospital IT, telehealth, and AI technologies in that order of importance. Third, there was a notable increase in positive attitudes towards AI technologies among students at both initial (t_0_) and follow-up (t_1_) assessments. Fourth, most participants expressed a desire to incorporate dHealth in their future medical practice, particularly in areas of patient communication, monitoring, follow-up, and in the prevention, diagnosis, and treatment of diseases.

**Table 2.** Comparison of medical students’ views and intentions (t_0_ vs t_1_)^a^.

Research construct and research variable	Pre–COVID-19, t_0_ (N=184), mean	Post–COVID-19, t_1_ (N=177), mean	*t* test (df)	*U* test (Mann-Whitney)
Individual background				
Age (years)	22.9	22.8	0.2 (359)	15,963
Gender	—^b^	—	—	U^c^
Academic level	2.9	2.4	3.6 (352) ^d^	13,021^d^
Experimentation with dHealth^e^ technologies				
Hospital IT systems	1.8	1.8	0.2 (359)	15,845
Telehealth applications	1.2	1.6	−6.3 (269)^d^	10,957^d^
AI-related^f^ technologies	1.3	1.5	−3.7 (325)^d^	12,978^d^
Mobile apps	1.4	1.4	0.9 (359)	16,106
Importance of dHealth in medical curriculum				
Hospital IT systems	4.1	4.2	−2.2 (359)^g^	14,446^h^
Telehealth applications	3.5	4.1	−6.9 (359)^d^	9441^d^
AI-related technologies	3.5	3.7	−1.8 (359)^h^	14,085^g^
Role of AI in the future of medicine				
For the medical profession	3.6	3.7	−2.1 (358)^g^	13,339^g^
For various medical specialties	3.4	3.5	−2.0 (359)^g^	13,885^g^
For one’s own medical practice	3.9	4.8	−2.6 (359)^g^	13,795^g^
Intent to integrate dHealth in medical practice				
Patient communication and consultation	3.4	4.2	−8.0 (250)^d^	9649^d^
Patient monitoring and follow-up	3.3	4.0	−6.8 (275)^d^	11,021^d^
Disease prevention, diagnosis, treatment	3.6	4.2	−5.5 (244)^d^	13,451^g^

a Adjustments for multiple hypothesis testing [38] are not required because no hypotheses are made as to differences between each of the 16 research variables at t_0_ versus t_1_.

b Not available.

c *χ*2=3.7 (*df*=2, *P*=.16).

d *P*<.001.

e dHealth: digital health.

f AI: artificial intelligence.

g *P*<.05.

h *P*<.10.

When comparing the 2 student groups (t_0_ and t_1_), *t* test analyses and Mann-Whitney (nonparametric) tests revealed significant differences in most of our research variables between t_0_ and t_1_. In the replication study (t_1_), students reported more experience with telehealth and AI technologies than in the initial survey (t_0_). However, their use of mobile apps and health IT did not change, potentially due to the reduced participation of fourth- and fifth-year students at t_1_ compared with t_0_. The heightened interest in telehealth and AI education at t_1_ could be attributed to the rise of telemedicine during the COVID-19 pandemic [39] and the increasing prominence of generative AI tools such as ChatGPT [40]. This may explain students’ increased intention to adopt dHealth in their medical practice.

### Variance Perspective Results

#### Measurement Model

The measurement instrument’s psychometric properties were assessed as a first step in the partial least squares–SEM analysis, confirming the reliability and validity of the instrument’s items and scales. The variance inflation factors of all our variables for both the structural and measurement models (inner model and outer model, respectively) are less than 2, showing that multicollinearity is not an issue in this study [32,41]. Reliability coefficients were all above the minimum acceptable of 0.60 for exploratory research [42]. Analysis of the external loadings shows that the indicators are all above 0.70, which supports an adequate representation of the indicators in their constructs (available upon request). Last, as evidence of discriminant validity, the square roots of the shared variance between constructs and their measures are shown to be higher than the correlations between constructs (available upon request).

#### Causal Analyses

##### Causal Analysis at t_0_

**Figure 2.**
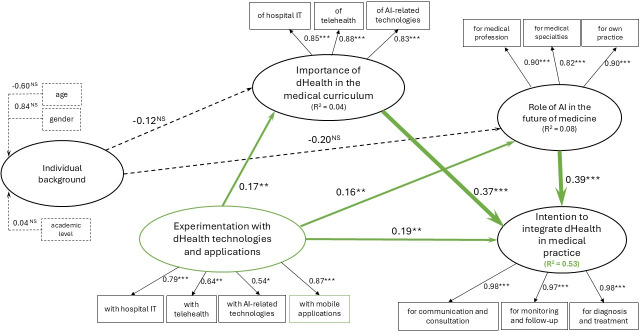
Pre–COVID-19 partial least squares results. AI: artificial intelligence; dHealth: digital health; NS: not significant (*P*>.10). *P<.10, **P<.05, ***P<.001.

Individual background is not significantly linked to the importance of dHealth and the role of AI in the future of medicine (Figure 2). These findings do not support H1 and H2 and suggest that medical students’ perceptions are more driven by factors related to TAMs than by demographic characteristics such as age, gender, or academic level. One of these factors is facilitating conditions, which is positively and significantly related to the importance of dHealth, role of AI in the future of medicine, and intention to integrate dHealth in medical practice. These results confirm H3, H4, and H5. Concerning the other 2 factors impacting Intention to integrate dHealth, namely Importance of dHealth in the curriculum and Role of AI in the future of medicine, we found that both have a positive and significant impact, confirming H6 and H7.

##### Causal Analysis at t_1_

**Figure 3.**
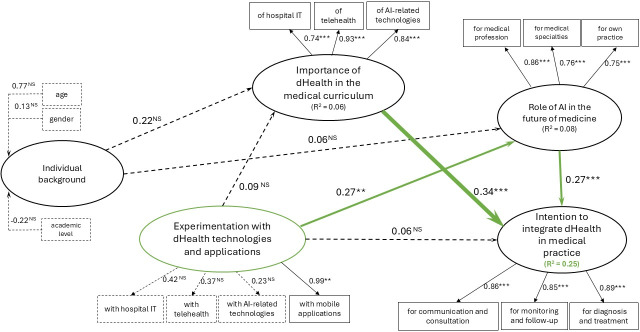
Post–COVID-19 partial least squares results. AI: artificial intelligence; dHealth: digital health; NS: not significant (*P*>.10). *P<.10, **P<.05, ***P<.001.

The relationships between individual background and both the importance of dHealth and the role of AI in medicine are not significant, contradicting H1 and H2 (Figure 3). Conversely, experimentation with dHealth is only positively and significantly related to the role of AI in the future of medicine. This confirms H4 but disconfirms H3 and H5. Additionally, the importance of dHealth in the curriculum and the role of AI in future medicine both exhibit a positive and significant influence on intention to integrate dHealth in future medical practice, confirming H6 and H7.

Globally, the relationships hypothesized in our model explain respectively 53% at t_0_ and 25% at t_1_ of the variance in medical students’ intention to integrate dHealth in medical practice (*R*^2^). Table 3 presents a comparative summary of the causal analysis results at t_0_ and t_1_.

**Table 3.** Comparative results at t_0_ and t_1_.

Hypothesis	Confirmation		Coefficient of causality	
	t_0_	t_1_	t_0_	t_1_
H1			−0.12^a^	0.22^a^
H2			−0.20^a^	0.06^a^
H3	✓		0.17^b^	0.09^a^
H4	✓	✓	0.16^b^	0.27^b^
H5	✓		0.19^b^	0.06^a^
H6	✓	✓	0.37^c^	0.34^c^
H7	✓	✓	0.39^c^	0.27^c^

a Not significant.

b *P*<.05.

c *P*<.001.

### Configurational Perspective Results

#### Configurational Model

Before conducting the configurational analysis (of sufficient conditions), we performed an analysis of necessary conditions with fsQCA to identify any univariate conditions essential for producing the outcome. An element is considered a candidate for a necessary condition if its set membership value is consistently equal to or higher than the set membership value in the outcome. We set a threshold of 0.90 consistency to determine necessity [34,43], though above 0.80 with equally high coverage can also be considered a candidate.

Before COVID, the role of AI in the future of medicine had the highest consistency (0.801) and coverage (0.887) for “High Intention” to integrate dHealth in medical practice. The importance of dHealth in the medical curriculum also showed high consistency (0.736) and coverage (0.705). Other elements, like experimentation with dHealth technology, academic level, and gender, had lower values. After COVID, experimentation with dHealth technologies and apps had the highest consistency (0.697) and coverage (0.755). The role of AI maintained high consistency (0.789) and coverage (0.854), while the importance of dHealth in the curriculum remained significant in terms of consistency (0.643) and coverage (0.778). Academic level and gender continued to show lower but consistent values compared with pre-COVID results. Detailed results of the analysis of necessary conditions are available in Multimedia Appendix 2. Overall, these results highlight that the significance of certain conditions, particularly the role of AI and the importance of dHealth, remained notable both before and after COVID. Additionally, the emphasis on experimentation with dHealth technologies and apps increased post-COVID, supporting the need for further analysis using fsQCA to explore complex causal relationships among these conditions.

For the configurational analysis with fsQCA, we examined the different combinations of Individual background, Importance of dHealth in the medical curriculum, Experimentation with dHealth technologies and apps, and Role of AI in the future of medicine, that is, the sufficient conditions for medical students to have a high intention to integrate dHealth in their future medical practice.

#### Pre–COVID-19 fsQCA Results

**Figure 4.**
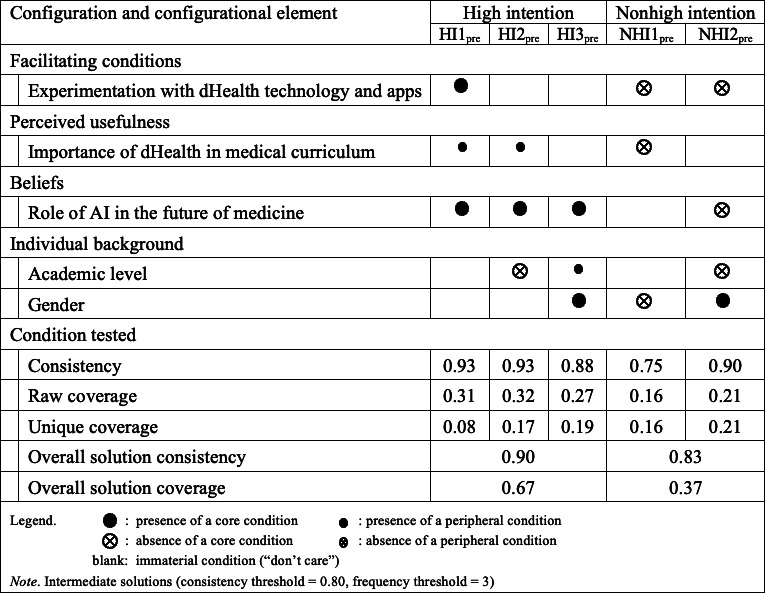
Configurations for the presence and absence of high intention to integrate dHealth in future medical practice at t_0_ (N=184). AI: artificial intelligence; dHealth: digital health.

The fsQCA results (Figure 4) show the configurations leading to high and nonhigh intentions to integrate dHealth in medical practice before COVID. The analysis reveals that before COVID (pre), the role of AI in the future of medicine consistently appears as a core condition across all high-intention configurations (HI1_pre_, HI2_pre_, HI3_pre_), with consistency values of 0.934, 0.927, and 0.884, respectively. Additionally, the importance of dHealth in the medical curriculum also plays a significant role, either as a core or peripheral condition, with relatively high consistency values of 0.736 and 0.705. Experimentation with dHealth technologies appears as a peripheral condition in all high-intention configurations, while academic level shows up as a peripheral condition in HI1_pre_ and HI3_pre_, but is absent in HI2_pre_.

For nonhigh intention configurations (NHI1_pre_ and NHI2_pre_), gender appears as a core condition in NHI1_pre_, with a consistency of 0.754, suggesting that it is a significant determinant of nonhigh intention. The importance of dHealth in the medical curriculum appears as a core condition in NHI2_pre_, with a consistency of 0.897, but in a different context compared with high intention configurations. The role of AI in the future of medicine appears as a peripheral condition in NHI2_pre_.

These results highlight the importance of beliefs about AI’s role in medicine for integrating dHealth practices. The consistent presence of the role of AI in high intention configurations indicates that such beliefs are crucial, indeed “necessary” for fostering high intentions to integrate dHealth in medical practice. The importance of dHealth in the curriculum and experimentation with technologies are also significant factors, suggesting that both educational emphasis and practical experience are vital. The appearance of gender as a core condition in non–high-intention configurations may reflect underlying biases or barriers that need to be addressed to improve dHealth integration. The overall solution consistency and coverage values indicate that the identified configurations are robust and provide substantial explanatory power for both high and nonhigh intentions to integrate dHealth in medical practice.

#### Post–COVID-19 fsQCA Results

**Figure 5.**
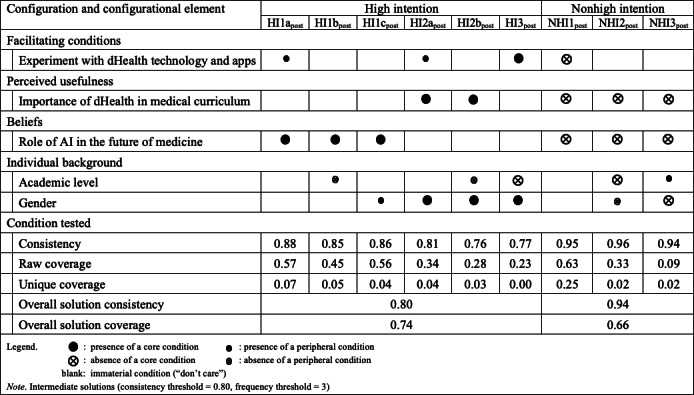
Configurations for the presence and absence of a high intention to integrate dHealth in future medical practice at t_1_ (N=177). AI: artificial intelligence; dHealth: digital health.

The after-COVID (post) configurational results are presented in Figure 5. For high-intention configurations (HI1a_post_, HI1b_post_, HI1c_post_, HI2a_post_, HI2b_post_, and HI3_post_), the most notable elements are the role of AI in the future of medicine and the importance of dHealth in the medical curriculum. The role of AI in the future of medicine appears consistently as a core condition in multiple configurations (HI1b_post_, HI1c_post_, and HI2b_post_), with consistency values ranging from 0.763 to 0.848. The importance of dHealth in the medical curriculum appears as both a core and peripheral condition in various configurations, with notable consistency values of 0.883 and 0.855. Experimentation with dHealth technologies is a core condition in the HI1a_post_ configuration, with a consistency of 0.883 and a raw coverage of 0.573.

For nonhigh intention configurations (NHI1_post_, NHI2_post_, and NHI3_post_), the role of AI in the future of medicine and the importance of dHealth in the medical curriculum also appear as core and peripheral conditions. The role of AI is a core condition in NHI2_post_, with a high consistency of 0.959 and a raw coverage of 0.627. Gender appears as a core condition in NHI1_post_, with a consistency of 0.947 and a raw coverage of 0.245.

These results highlight that the significance of certain conditions, particularly the role of AI and the importance of dHealth in the curriculum, remained notable after COVID. The emphasis on experimentation with dHealth technologies and apps also remains significant for high-intention configurations. The appearance of gender as a core condition in non–high-intention configurations suggests potential underlying biases or barriers that need to be addressed. The overall solution consistency and coverage values indicate that the identified configurations provide substantial explanatory power for both high- and non–high-intentions to integrate dHealth in medical practice. This supports the need for targeted interventions that emphasize the role of AI and practical experimentation with dHealth technologies to foster higher intentions to integrate dHealth in medical practice.

## Discussion

### Main Findings

This study offers a detailed account of why medical students are inclined to integrate dHealth technologies into their future medical practice. It was observed that respondents, particularly those in their early years of medical education, have had limited opportunities to engage with dHealth technologies. These limited interactions suggest a persistent disconnect between students’ educational experience and the digital demands of clinical environments. This misalignment calls for a revaluation of curriculum design, particularly in the preclinical years, where exposure to digital tools remains insufficient.

While our data show an increasing engagement with AI and other digital tools post-COVID, these changes may reflect not only pandemic adaptations but also broader societal transformations. Rather than attributing the improvement solely to the pandemic, it is likely that these shifts are part of a broader evolution in health care, driven by rapid technological advancements [44].

Our analysis further revealed that factors such as age, gender, and academic level did not show statistically significant associations with the adoption of dHealth technologies among medical students. This challenges the assumption that demographic attributes are key determinants of digital readiness. Instead, students’ belief in the role of AI and perceived usefulness of dHealth emerged as salient antecedents. This suggests that cultivating positive attitudes and beliefs through interventions, such as AI literacy modules or simulations, may be more effective than broad curriculum reforms.

Our findings further underscore the imperative of integrating digital transformation strategies within medical education. According to Matt et al [2], successful digital transformation involves strategic considerations across 4 dimensions: technological utilization, value creation changes, structural modifications, and financial aspects. Our results reveal that medical students recognize the enhanced value that dHealth technologies bring to health care delivery, highlighting a shift towards more patient-centered and technologically integrated care processes. Moreover, as digital health care continues to evolve, medical schools must adapt their training environments, ensuring that students acquire relevant digital competencies. Financial aspects are also pivotal, as the increasing demand for digital competencies suggests that investments in educational resources and faculty training are necessary. By aligning dHealth curriculum enhancements with these transformational dimensions, medical schools can better prepare future physicians to navigate and lead in an increasingly digital health care landscape.

### Study Contributions and Implications

Our results outline the importance of enhancing future physicians’ digital literacy, focusing on key dHealth tools such as electronic medical records, clinical decision systems, AI-based tools, basics of programming and cybersecurity, and telehealth. Future doctors should also experience how patient data tracking tools help in managing health conditions and communication with health professionals, among others. Medical schools in Canada and abroad are encouraged to form groups including professors, students, and patients to periodically review dHealth training needs. Promoting hands-on experience with current and emerging technologies, starting from the preparatory year, is recommended [45]. Medical schools could also facilitate interdisciplinary collaboration through events like hackathons [46,47].

While there are abundant opportunities to integrate dHealth in the medical curriculum, we concur with Aungst and Patel [48] that, most often, the availability of faculty and experts to facilitate learning experiences does not match present needs and requirements in this regard. Integrating dHealth topics into the medical curriculum thus poses challenges to educators, leading many medical schools to consider providing specialized tracks, certificates, or potentially adjunctive degrees for interested students. To facilitate such developments, dHealth tracks and programs could be created in partnership with other faculties, such as engineering, computer science, and business schools.

Our findings suggest that medical education must adapt to shifting dynamics in dHealth, recognizing that multiple factors, including technological advancements and increased reliance on AI tools, are shaping students’ perceptions and intentions. Emphasizing practical experimentation with dHealth technologies should become a priority, reflecting its growing significance in medical training. Additionally, addressing underlying biases, such as the significant role of gender in non–high-intention configurations, should be part of the strategy to foster a more inclusive approach to dHealth integration. Medical schools must remain agile, continually updating curricula to reflect these developments, and consider more flexible and interdisciplinary training programs that can better prepare future physicians for the evolving dHealth landscape. By adopting a forward-looking approach to dHealth education, institutions can ensure that future physicians are equipped with the necessary competencies to navigate and lead in an increasingly digital health care environment.

While our study was conducted in Quebec, where the health care system is publicly administered and undergoing a substantial digital transformation (eg, with the ongoing rollout of the EPIC EHR system in major institutions), dHealth education in medical faculties remains uneven and largely decentralized. This may help explain the limited exposure to dHealth technologies reported by students in our sample. Quebec’s case reflects both the opportunities and the challenges of aligning large-scale digital infrastructure investments with educational reform. Although specific features of the Quebec context may not be fully generalizable, the broader patterns we observed, such as the importance of perceived usefulness and the growing influence of AI, are likely to resonate with medical schools in other regions undergoing similar transitions. A more context-sensitive approach to curricular reform may thus be warranted.

It is also important to interpret our findings in light of the specific context of medical education in Quebec. Unlike other provinces in Canada and many international settings, Quebec offers a direct-entry medical program following CEGEP, which means students often begin their training at a younger age and with less prior academic or clinical exposure. This structure may limit early opportunities to engage with dHealth tools unless intentionally built into the curriculum. Furthermore, dHealth education in Quebec remains decentralized, with substantial variation across faculties and no province-wide mandate or standardized framework for integrating digital competencies. This contrasts with efforts in some US and European medical schools, where formal dHealth tracks, AI literacy modules, or interdisciplinary partnerships have been implemented. These contextual differences may partly explain the limited dHealth exposure reported by our respondents and highlight the importance of tailoring curricular innovations to local structural and institutional realities.

### Study Limitations and Suggestions for Future Research

The findings of this study should be interpreted with caution in light of certain limitations. For one thing, the study was conducted at a single medical school and achieved relatively low response rates, which may constrain the generalizability of the results to other contexts. Nonetheless, the institution admits a large and socio-demographically diverse student body, and the sample included participants across multiple training stages, thereby capturing a range of perspectives. Moreover, the study’s primary objective was not to estimate population-level prevalence but to examine theoretically informed relationships among key constructs. From this perspective, the observed associations remain analytically robust and informative. Future research could build on these findings by investigating dHealth education across medical schools in Canada and internationally, enabling comparisons of students’ dHealth proficiency, attitudes, and intentions.

Next, our research model is based on 2 theoretical foundations. Future research could expand this model to include factors like social influence and effort expectancy [22] as well as IT-enabled medical knowledge management capabilities [49]. Additionally, we acknowledge that other individual differences, such as students’ specialization choices and prior clinical experience in different medical settings (eg, intensive care units vs outpatient clinics), may influence their perceptions and adoption of dHealth technologies. While our study did not control for these variables, future research could explore their impact to provide a more nuanced understanding of how diverse clinical exposures shape students’ intentions to integrate dHealth into their practice. Moreover, while our study focuses on medical students, these findings may also offer insights into how other health care professionals have developed new perceptions of dHealth. Although students have more limited exposure to these tools compared with practicing physicians, the shifts in their attitudes may reflect broader trends in the medical field. Understanding these evolving perceptions could help anticipate how dHealth adoption progresses across different health care professions.

We also encourage researchers to gather qualitative data through interviews or focus groups to enhance our comprehension of medical students’ and physicians’ motivations, concerns, and experiences with dHealth technologies. Beyond the possible influence of the COVID-19 pandemic [50,51], the increasing use of dHealth technologies has raised concerns about digital fatigue [52]. Future research could explore the tipping point at which the use of dHealth technologies overwhelms medical practitioners, adversely affecting their performance. Additionally, investigating how AI shapes the use of evidence-based medicine presents another promising avenue for future research.

Another limitation of this study relates to the content validity of the measure of the Experimentation with dHealth technologies and the Importance of dHealth in the medical curriculum constructs. While sufficient to fully answer our research questions and rigorously test our theoretical model, the content validity of these 2 measures could be increased by replacing one “general” measurement item, such as one related to machine learning or big data, with multiple “specific” items delineating particular technologies or apps. This may also entail measurement scales that are more specific to each technology and app than the general scales presently used. However, such a measurement approach would have significantly increased the size and complexity of the survey questionnaire. Future studies should seek to improve these measures by gaining a deeper understanding of how medical students specifically encounter dHealth technologies and apps in the course of their curriculum.

Also warranting further exploration, the fsQCA results reveal notable differences in the configurations leading to high and nonhigh intentions to integrate dHealth in medical practice before and after COVID. Before COVID, the role of AI in the future of medicine consistently appeared as a core condition in high-intention configurations, with the highest consistency and coverage values. The importance of dHealth in the medical curriculum also played a significant role, either as a core or peripheral condition, alongside experimentation with dHealth technologies. In contrast, post-COVID results indicate a shift in emphasis. Experimentation with dHealth technology emerges as a core condition in one of the high-intention configurations, reflecting increased importance in the later period. While the role of AI and the importance of dHealth remain significant, their roles are slightly reconfigured, with AI appearing as a core condition in multiple high-intention configurations, and the importance of dHealth appearing as both a core and peripheral role.

Last, gender appears as a significant core condition in nonhigh intention configurations both pre- and post-COVID, suggesting persistent underlying biases. The overall solution consistency and coverage values are slightly higher post-COVID for non–high-intention configurations, indicating a stronger explanatory power of the identified configurations in the later period. These last results warrant further study of the evolving nature of the factors influencing the integration of dHealth practices, emphasizing the increasing importance of practical experimentation and persistent beliefs about AI’s role in medicine.

### Conclusions

This study investigates the factors influencing medical students’ intentions to integrate dHealth technologies into their future practice and examines changes in their perceptions over time. We used a 2-phase survey at a large Canadian medical school to assess changes in perceptions before and after the COVID-19 pandemic. Our findings indicate limited exposure to dHealth technologies within the medical curriculum. However, there was a strong consensus on the necessity of formal dHealth training. A notable increase in acceptance of AI and telehealth tools was observed, reflecting broader technological advancements and shifts in dHealth practices.

Our work underscores the need for curriculum reforms to ensure medical students gain practical exposure to dHealth technologies. While dHealth tools are becoming increasingly central to medical practice, concerns such as digital fatigue and the importance of hands-on training must be considered in designing effective educational programs. Medical schools must enhance their educational strategies and resources to keep pace with the evolving landscape of dHealth, ensuring that future physicians are well-prepared to navigate and integrate digital technologies effectively in clinical environments.

## Supplementary material

10.2196/64804 Multimedia Appendix 1 Survey items.

10.2196/64804 Multimedia Appendix 2 Analysis of necessary configurational elements.

## Abbreviations

AI: artificial intelligence
CMB: common method bias
dHealth: digital health
EHR: electronic health record
fsQCA: fuzzy-set qualitative comparative analysis
IS: information system
SEM: structural equation modeling
TAM: technology acceptance model
