# Supplementary material for: Evolving Medical Students’ Digital Health Perceptions and Intentions: Insights From a Prepandemic and Postpandemic Survey Study
Source: J Med Internet Res. 2025 Sep 3;27:e64804. doi: 10.2196/64804 (PMC12407568; doi:10.2196/64804)
Supplement: Multimedia Appendix 1 [file jmir-v27-e64804-s001.docx]

Q1.1 What year of training are you currently in?

- Preparatory year
- First year preclinical
- Second year preclinical
- First year clerkship
- Second year clerkship

Q1.2 What is your gender?

- Female
- Male
- Prefer not to reply

Q2.1. How familiar are you with each of the following technologies and applications?

|  | Very low | Low | Moderate | High | Very high |
| --- | --- | --- | --- | --- | --- |
| Electronic medical record (DMÉ) |  |  |  |  |  |
| Computerized clinical record (DCI) |  |  |  |  |  |
| Dossier santé Québec (DSQ) |  |  |  |  |  |
| Carnet santé Québec |  |  |  |  |  |
| Portal « Rendez-vous santé Québec » |  |  |  |  |  |
| Telehealth |  |  |  |  |  |
| Tele-expertise |  |  |  |  |  |
| Artificial Intelligence |  |  |  |  |  |
| Machine learning |  |  |  |  |  |
| Big data |  |  |  |  |  |
| Internet of things |  |  |  |  |  |

|  |  |
| --- | --- |

Q2.2. To what extent have you been exposed to each of the following technologies and applications during your medical training (courses and/or internships)?

|  | None | A little bit | Moderately | Quite a bit | Very |
| --- | --- | --- | --- | --- | --- |
| Electronic medical record (DMÉ) |  |  |  |  |  |
| Computerized clinical record (DCI) |  |  |  |  |  |
| Dossier santé Québec (DSQ) |  |  |  |  |  |
| Carnet santé Québec |  |  |  |  |  |
| Portal « Rendez-vous santé Québec » |  |  |  |  |  |
| Telehealth |  |  |  |  |  |
| Tele-expertise |  |  |  |  |  |
| Artificial Intelligence |  |  |  |  |  |
| Machine learning |  |  |  |  |  |
| Big data |  |  |  |  |  |
| Internet of things |  |  |  |  |  |

Q2.3. How familiar are you with each of the following connected medical devices?

|  | None | A little bit | Moderately | Quite a bit | Very |
| --- | --- | --- | --- | --- | --- |
| Connected sphygmomanometer |  |  |  |  |  |
| Connected saturometer |  |  |  |  |  |
| Connected thermometer |  |  |  |  |  |
| Connected glucose meter |  |  |  |  |  |
| Connected ophthalmoscope |  |  |  |  |  |
| Connected autorefractometer |  |  |  |  |  |
| Connected miniature electrocardiogram |  |  |  |  |  |
| Connected otoscope |  |  |  |  |  |
| Connected spirometer |  |  |  |  |  |
| Connected stethoscope |  |  |  |  |  |
| Connected ultrasound probe |  |  |  |  |  |

Q3.1. How often do you use each of the following mobile applications as part of your medical training?

|  | Never | Rarely | Regularly | Frequently | Very frequently |
| --- | --- | --- | --- | --- | --- |
| UpToDate (1) |  |  |  |  |  |
| BMJBestPractice (2) |  |  |  |  |  |
| Calculate by QxMD (3) |  |  |  |  |  |
| ClinicalKey (4) |  |  |  |  |  |
| DxSaurus (5) |  |  |  |  |  |
| DynaMed Mobile (6) |  |  |  |  |  |
| Epocrates (7) |  |  |  |  |  |
| INESSS (8) |  |  |  |  |  |
| IPharmacy (9) |  |  |  |  |  |
| Lanthier (10) |  |  |  |  |  |
| MDCalc (11) |  |  |  |  |  |
| MedCalx (12) |  |  |  |  |  |
| MedPage Today (13) |  |  |  |  |  |
| Medscape (14) |  |  |  |  |  |
| NEJM This Week (15) |  |  |  |  |  |
| Omnio (16) |  |  |  |  |  |
| Pepid (17) |  |  |  |  |  |
| Autre : (18) |  |  |  |  |  |

Q4.1. In your opinion, to what extent will artificial intelligence and its components (machine learning, deep learning, etc.) have an effect on each of the following aspects of the medicine of the future?

|  | Very negative effect | Negative effect | No effect | Positive effect | Very positive effect |
| --- | --- | --- | --- | --- | --- |
| Disease prevention |  |  |  |  |  |
| Disease diagnosis |  |  |  |  |  |
| Disease treatment |  |  |  |  |  |
| Disease prognosis |  |  |  |  |  |
| Patient-physician relationship |  |  |  |  |  |

Q4.2. To what extent do you think each of the medical specialties below will be affected by artificial intelligence in the future?

|  | None | A little bit | Moderately | Quite a bit | Very |
| --- | --- | --- | --- | --- | --- |
| Pathology |  |  |  |  |  |
| Radiology |  |  |  |  |  |
| Dermatology |  |  |  |  |  |
| Ophthalmology |  |  |  |  |  |
| Emergency and critical care |  |  |  |  |  |
| Family medicine |  |  |  |  |  |
| Internal medicine |  |  |  |  |  |
| Psychiatry |  |  |  |  |  |
| Surgery |  |  |  |  |  |

Q4.3. Do you intend to use artificial intelligence once you have completed your studies to carry out the following tasks in your medical practice?

|  | Yes | No |
| --- | --- | --- |
| Analyze radiological images |  |  |
| Analyze photographic images (e.g. fundus) |  |  |
| Analyze pathological images (e.g. biopsy specimens) |  |  |
| Diagnose patients |  |  |
| Patient prognosis |  |  |
| Determine patient management protocols |  |  |
| Analyze the data from my medical history to generate an opinion |  |  |
| Supervise and evaluate patient interviews |  |  |

Q5.1 To what extent do you adopt the practices listed below as part of your medical training?

|  | Never | Rarely | Regularly | Frequently | Very frequently |
| --- | --- | --- | --- | --- | --- |
| I read the scientific literature to enhance my medical training, beyond the mandatory texts. |  |  |  |  |  |
| I search the web to identify relevant and credible sources that can contribute to my medical training. |  |  |  |  |  |
| I'm on the lookout for medical practice guidelines |  |  |  |  |  |
| I take a critical look at scientific and professional literature related to medicine |  |  |  |  |  |
