# Supplementary material for: Evolving Medical Students’ Digital Health Perceptions and Intentions: Insights From a Prepandemic and Postpandemic Survey Study
Source: J Med Internet Res. 2025 Sep 3;27:e64804. doi: 10.2196/64804 (PMC12407568; doi:10.2196/64804)
Supplement: Multimedia Appendix 2 [file jmir-v27-e64804-s002.docx]

Table S1. Analysis of necessary configurational elements (${n=184, t}_{pre-Covid}$)

| Configurational element | High Intention^a^ | | Non-High Intention^b^ | |
| --- | --- | --- | --- | --- |
|  | Consistency | Coverage | Consistency | Coverage |
| **Facilitating Conditions** |  |  |  |  |
| Experimentation with dHealth tech. & apps | 0.447 | 0.679 | 0.272 | 0.331 |
| **Perceived Usefulness** |  |  |  |  |
| Importance of dHealth in med. curriculum | 0.736 | 0.705 | 0.492 | 0.378 |
| **Beliefs** |  |  |  |  |
| Role of AI in the future of medicine | 0.801 | 0.887 | 0.274 | 0.243 |
| **Individual Background** |  |  |  |  |
| Academic level | 0.584 | 0.553 | 0.590 | 0.447 |
| Gender^c^ | 0.620 | 0.532 | 0.680 | 0.468 |

^a^ calibration: [fully-in = top quartile, crossover = median, fully-out = bottom quartile]

^b^ negated set (~)

^c^ crisp set: [fully-in = 1, fully-out = 0]

Table S2. Analysis of necessary configurational elements (n = 177, $t_{post-Covid}$)

| Configurational element | High Intention^a^ | | Non-High Intention^b^ | |
| --- | --- | --- | --- | --- |
|  | Consistency | Coverage | Consistency | Coverage |
| **Facilitating Conditions** |  |  |  |  |
| Experimentation with dHealth tech. & apps | 0.697 | 0.755 | 0.388 | 0.375 |
| **Perceived Usefulness** |  |  |  |  |
| Importance of dHealth in med. curriculum | 0.643 | 0.778 | 0.314 | 0.338 |
| **Beliefs** |  |  |  |  |
| Role of AI in the future of medicine | 0.789 | 0.854 | 0.290 | 0.280 |
| **Individual Background** |  |  |  |  |
| Academic level | 0.434 | 0.575 | 0.360 | 0.425 |
| Gender^c^ | 0.717 | 0.541 | 0.700 | 0.471 |

^a^ calibration: [fully-in = top quartile, crossover = median, fully-out = bottom quartile]

^b^ negated set (~)

^c^ crisp set: [fully-in = 1, fully-out = 0]
